# Synergistic Effects of Water Management and Silicon Foliar Spraying on the Uptake and Transport Efficiency of Cadmium in Rice (*Oryza sativa* L.)

**DOI:** 10.3390/plants12061414

**Published:** 2023-03-22

**Authors:** Xiaoyun Huang, Chengwu Fan, Dongyi Xie, Hongxing Chen, Song Zhang, Hui Chen, Song Qin, Tianling Fu, Tengbing He, Zhenran Gao

**Affiliations:** 1 College of Agriculture, Guizhou University, Guiyang 550025, China; 2 Institute of New Rural Development, Guizhou University, Guiyang 550025, China; 3 Guizhou Institute of Soil and Fertilizer, Guizhou Academy of Agricutural Science, Guiyang 550025, China

**Keywords:** water management, foliar spraying, Si, cadmium, photosynthesis

## Abstract

To study the synergistic effects of water management and silicon (Si) foliar spraying on the uptake and transport of cadmium (Cd) in rice, we designed four treatments: conventional intermittent flooding + no Si foliar spraying (CK), continuous flooding throughout the growth stage + no Si foliar spraying (W), conventional intermittent flooding + Si foliar spraying (Si) and continuous flooding throughout the growth stage + Si foliar spraying (WSi). The results show that WSi treatment reduced the uptake and translocation of Cd by rice and significantly reduced the brown rice Cd content, with no effect on rice yield. Compared with CK, the Si treatment increased the net photosynthetic rate (Pn), stomatal conductance (Gs) and transpiration rate (Tr) of rice by 6.5–9.4%, 10.0–16.6% and 2.1–16.8%, respectively. The W treatment decreased these parameters by 20.5–27.9%, 8.6–26.8% and 13.3–23.3%, respectively, and the WSi treatment decreased them by 13.1–21.2%, 3.7–22.3% and 2.2–13.7%, respectively. The superoxide dismutase (SOD) and peroxidase (POD) activity decreased by 6.7–20.6% and 6.5–9.5%, respectively, following the W treatment. Following the Si treatment, SOD and POD activity increased by 10.2–41.1% and 9.3–25.1%, respectively, and following the WSi treatment, they increased by 6.5–18.1% and 2.6–22.4%, respectively. Si foliar spraying ameliorated the detrimental effects of continuous flooding throughout the growth stage on photosynthesis and antioxidant enzyme activity. We conclude that synergistic continuous flooding throughout the growth stage, combined with Si foliar spraying, can significantly block Cd uptake and translocation and is therefore an effective means of reducing the accumulation of Cd in brown rice.

## 1. Introduction

Cadmium (Cd) is a biotoxic environmental heavy metal with high water solubility, mobility and persistence [1]. Approximately 7% of China’s land is contaminated with Cd [2]. Cd accumulation reduces crop photosynthesis, increases the peroxidation of cell membrane lipids and inhibits the activity of antioxidant enzymes [3,4], thereby inhibiting normal crop growth and reducing yields. Rice is the world’s largest planted crop and is a staple food for an estimated three billion people [5]. Rice is highly susceptible to Cd [6], which it readily absorbed, entering the human body via the food chain and leading to health risks [7]. Thus, determining how to effectively reduce Cd accumulation in brown rice is of particular important to ensure safety in rice production and human health, as well as sustainable agricultural development.

At present, passivation materials such as lime, sea foam and biochar can effectively reduce Cd accumulation in rice [8]. However, not only are these passivation materials expensive, but their long-term application can also reduce the effect of passivation, damage the soil structure and even cause secondary contamination [9]. Water management is a more cost-effective and environmentally friendly measure compared to passivation materials [10,11]. Water management during the rice growth stage can greatly affect its plant growth, regulate Cd activity in soil and modify Cd accumulation in rice seeds [12]. Some studies have shown that, compared to conventional intermittent flooding, continuous flooding throughout the growth stage can reduce soil Cd solubility [13], inhibiting the formation of iron films on the root surface of rice [14], reducing its uptake of Cd. However, continuous flooding may also reduce chlorophyll content in rice leaves, leading to a reduction in the net rate of photosynthesis (Pn) in rice yield [15]. Thus, measures are urgently required to reduce Cd content while ensuring rice yields.

Silicon (Si) foliar spraying has been shown to be an effective means of reducing Cd accumulation in rice [16]. In addition to reducing Cd uptake, Si foliar spraying does not reduce rice yield [17]. Si is the most abundant element after oxygen in the Earth’s crust and overlying soil [18]. Si is beneficial for rice and is even considered an essential nutrient [19]. Si foliar spraying not only mitigates the detrimental effects of Cd on rice growth and enhances the antioxidant defense system [20] but also enhances photosynthesis in rice leaves and reduces Cd transport from the shoots to brown rice [21]. While Si foliar spraying can reduce the Cd content of brown rice without affecting rice yield, its Cd reduction effect is not as significant as that of continuous flooding treatment.

In summary, a single measure cannot simultaneously take into account the effects on Cd reduction and rice yield. Therefore, it is necessary to investigate the effects of different synergistic measures of water management and Si foliar spraying on Cd accumulation in rice. For this reason, we designed a pot experiment with two types of water management (conventional intermittent flooding and continuous flooding throughout the growth stage) and two types of Si foliar spraying treatment (no Si foliar spraying and Si foliar spraying). We explored the synergistic effects of these treatments on rice growth and yield and their combined effects on Cd uptake and accumulation in rice, with the aim of providing theoretical support to effectively reduce Cd accumulation in brown rice.

## 2. Results

### 2.1. Synergistic Effects of Water Management and Silicon Foliar Spraying on Cd Content in Rice Organs and Yield

**Figure 1:**
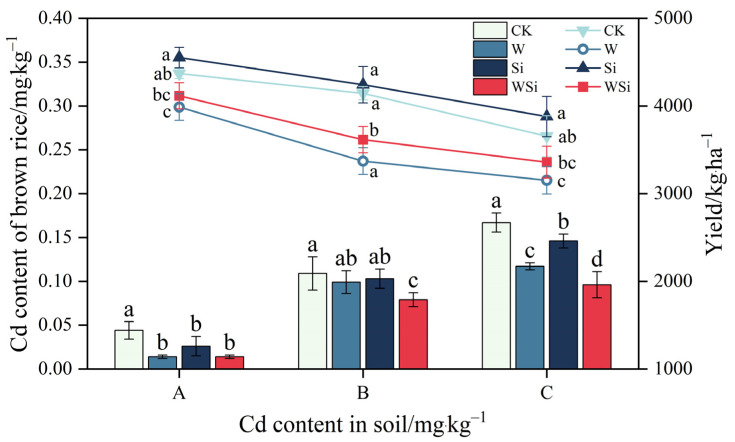
Synergistic effects of water management and silicon foliar spraying on Cd content of brown rice and rice yield. The histogram and line chart represent Cd content of brown rice and rice yield, respectively. The error bar represents three repeated standard deviations (SD). There is no significant difference between values with the same letters in the table (*p* ≥ 0.05, Fisher’s LSD test). A, B and C represent the actual measured Cd content in soil after the addition of exogenous Cd at 0.20 mg·kg^−1^, 0.60 mg·kg^−1^ and 1.60 mg·kg^−1^, respectively.

The synergistic effects of water management and silicon foliar spraying on the Cd content in rice organs and on rice yield are shown in Table 1 and Figure 1. The Cd content of rice organs increased with increasing Cd concentration, and rice yield decreased with increasing Cd concentration at all three Cd concentrations. At concentration A, the W, Si and WSi treatments all significantly increased the Cd content in the roots and leaves and significantly decreased the Cd content in brown rice compared to the CK treatment. They increased the Cd content in the roots by 37.0%, 112.8% and 68.3% (*p* < 0.05), increased the Cd content in the leaves by 50.8%, 32.0% and 87.8% (*p* < 0.05) and reduced the Cd content in brown rice by 67.1%, 42.1% and 68.5% (*p* < 0.05), respectively. The W and WSi treatments significantly reduced the Cd content in the stems by 47.9% and 44.7% (*p* < 0.05), respectively. Si treatment significantly increased the Cd content in the cob by 49.1% (*p* < 0.05) and reduced the Cd content in the husks by 29.5% (*p* < 0.05). The W treatment significantly reduced rice yield by 8.7% compared to the CK treatment (*p* < 0.05).

At concentration B, the WSi treatment significantly increased the Cd content in the roots by 62.2% (*p* < 0.05) and significantly reduced the Cd content in the brown rice by 26.9% (*p* < 0.05) compared to the CK treatment. The Si and WSi treatments significantly increased the Cd content in the stems by 146.5% and 120.2% (*p* < 0.05) and significantly increased the Cd content in the cob by 61.8% and 60.9% (*p* < 0.05), respectively. The W, Si and WSi treatments significantly reduced the Cd content in the husk by 36.0%, 41.0% and 39.6%, respectively (*p* < 0.05). Both the W and WSi treatments resulted in significant reductions in rice yield of 18.7% and 12.8%, respectively (*p* < 0.05).

At concentration C, the W treatment significantly reduced the Cd content in the roots by 29.0% compared to the CK treatment (*p* < 0.05). Both the W and WSi treatments significantly reduced the Cd content in the stems by 70.4% and 61.5%, respectively (*p* < 0.05). The W treatment resulted in a significant reduction in Cd content of 20.1% and 19.9% in the leaves and cob, respectively (*p* < 0.05). Si treatment significantly increased the Cd content in the leaves and cob by 26.4% and 62.8%, respectively (*p* < 0.05). The W, Si and WSi treatments resulted in a significant reduction in Cd content in the husks and brown rice and reduced the Cd content in the husks by 54.4%, 33.8% and 56.0% (*p* < 0.05) and in the brown rice by 29.7%, 12.6% and 33.8% (*p* < 0.05), respectively. The W treatment resulted in a significant 13.8% (*p* < 0.05) reduction in rice yield compared to the CK treatment.

Under the three Cd treatments, W, Si and WSi all reduced the Cd content in brown rice, with the largest reduction occurring under the WSi treatment, followed by the W treatment, with the smallest reduction occurring under the Si treatment. Furthermore, all W treatments significantly reduced rice yield.

### 2.2. Synergistic Effects of Water Management and Silicon Foliar Spraying on the Cd Translocation Factor of Rice Organs

**Figure 2:**
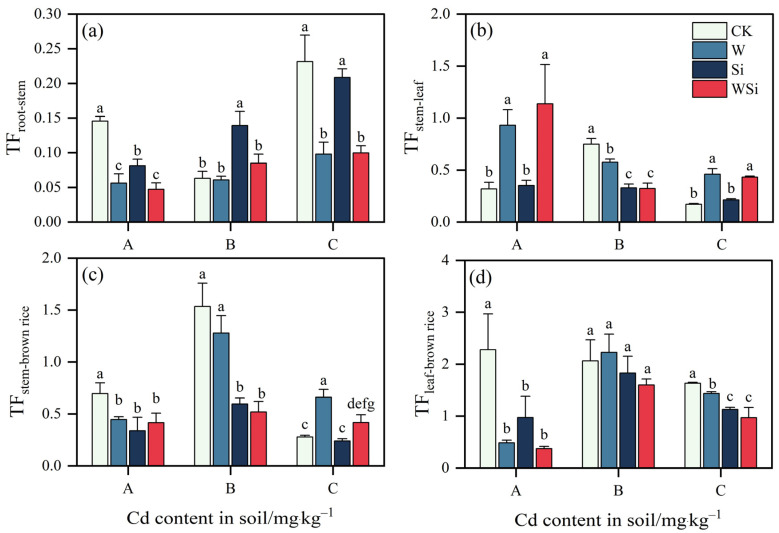
Synergistic effects of water management and silicon foliar spraying on the Cd translocation factor of rice organs ((**a**) TF_root−stem_; (**b**) TF_stem−leaf_; (**c**) TF_stem−brown rice_; (**d**) TF_leaf−brown rice_). The error bar represents three repeated standard deviations (SDs). There is no significant difference between values with the same letters in the table (*p* ≥ 0.05, Fisher’s LSD test). A, B and C represent the actual measured Cd content in soil after the addition of exogenous Cd at 0.20 mg·kg^−1^, 0.60 mg·kg^−1^ and 1.60 mg·kg^−1^, respectively.

The synergistic effects of the water management and silicon foliar spraying treatments on the Cd translocation factor of rice organs are shown in Figure 2. Compared with the CK treatment, at concentration A, the W, Si and WSi treatments all significantly reduced TF_root−stem_, TF_stem−brown_ and TF_leaf−brown rice_, reducing TF_root−stem_ by 61.5%, 44.1% and 67.5% (*p* < 0.05); TF_stem−brown rice_ by 35.9%, 51.4% and 40.3% (*p* < 0.05); and TF_leaf−brown rice_ by 78.7%, 57.3% and 83.5% (*p* < 0.05), respectively. The W and WSi treatments resulted in a significant increase in TF_stem−leaf_ of 192.5% and 257.2%, respectively (*p* < 0.05).

At concentration B, the Si treatment significantly increased TF_root−stem_ by 121.0% relative to the CK treatment. The W, Si and WSi treatments all significantly reduced TF_stem−leaf_ by 23.2%, 56.1% and 57.0%, respectively (*p* < 0.05). The Si and WSi treatments significantly reduced TF_stem−brown rice_ by 61.2% and 66.2%, respectively (*p* < 0.05). However, the W, Si and WSi treatments had no significant effect on TF_leaf−brown rice_.

At concentration C, both the W and WSi treatments significantly reduced TF_root−stem_ and increased TF_stem−leaf_ and TF_stem−brown rice_ relative to the CK treatment. They reduced TF_root−stem_ by 57.7% and 57.0% (*p* < 0.05), increased TF_stem−leaf_ by 170.7% and 153.9% (*p* < 0.05) and increased TF_stem−brown rice_ by 138.1% and 50.3%, respectively (*p* < 0.05). The W, Si and WSi treatments significantly reduced TF_leaf−brown rice_ by 12.0%, 30.9% and 40.6%, respectively (*p* < 0.05).

With the increase in Cd concentration, Cd transport from root to stem increased, and its transport from stem to leaf decreased. There were differences in the Cd transport coefficient of rice organs under the three different Cd concentrations, which explains the significant decrease in Cd in brown rice.

### 2.3. Synergistic Effects of Water Management and Silicon Foliar Spraying on SPAD Values of Rice Leaves

**Figure 3:**
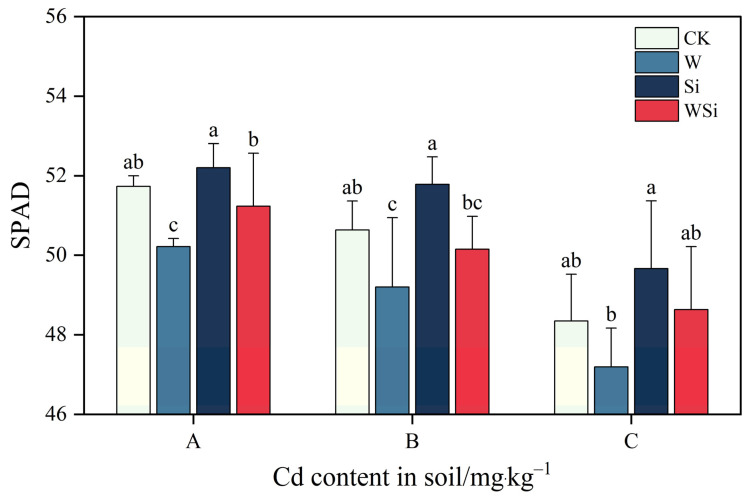
Synergistic effects of water management and silicon foliar spraying on SPAD values of rice leaves. The error bar represents three repeated standard deviations (SDs). There is no significant difference between values with the same letters in the table (*p* ≥ 0.05, Fisher’s LSD test). A, B and C represent the actual measured Cd content in soil after the addition of exogenous Cd at 0.20 mg·kg^−1^, 0.60 mg·kg^−1^ and 1.60 mg·kg^−1^, respectively.

The effects of different water management and Si foliar spraying treatments on the soil and plant analyzer development (SPAD) values of rice leaves are shown in Figure 3. The SPAD values decreased gradually with increasing Cd concentration. At concentrations A and B, the W treatment resulted in a significant reduction in SPAD values of 2.9% and 2.8%, respectively (*p* < 0.05). The Si treatment significantly increased the SPAD values at all three Cd concentrations relative to the W treatment. The WSi treatment also significantly increased the SPAD values in rice compared to the W treatment at concentration A. The W treatment decreased the SPAD values, the Si treatment increased the SPAD values and the WSi treatment decreased the SPAD values but increased them relative to the W treatment.

### 2.4. Synergistic Effects of Water Management and Silicon Foliar Spraying on Rice Photosynthesis

**Figure 4:**
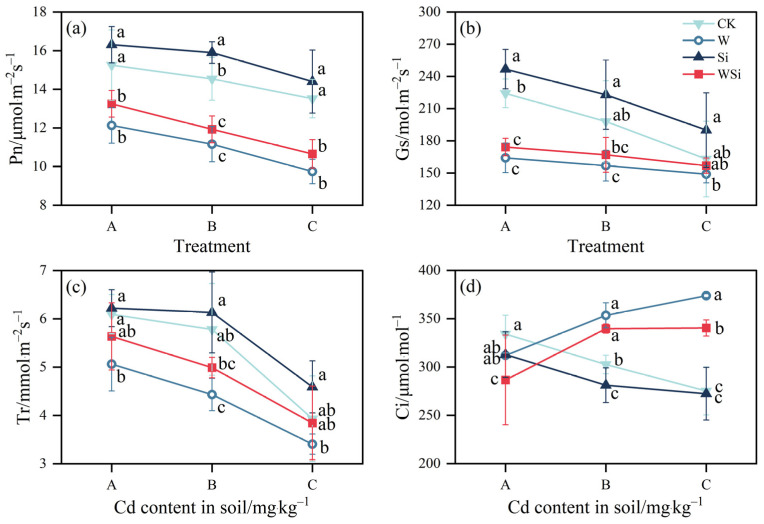
Synergistic effects of water management and silicon foliar spraying on rice photosynthesis ((**a**) net photosynthetic rate (Pn); (**b**) stomatal conductance (Gs); (**c**) transpiration rate (Tr); (**d**) intercellular CO_2_ concentration (Ci)). The error bar represents three repeated standard deviations (SDs). There is no significant difference between values with the same letters in the table (*p* ≥ 0.05, Fisher’s LSD test). A, B and C represent the actual measured Cd content in soil after the addition of exogenous Cd at 0.20 mg·kg^−1^, 0.60 mg·kg^−1^ and 1.60 mg·kg^−1^, respectively.

The synergistic effects of water management and silicon foliar spraying on the photosynthesis parameters of rice are shown in Figure 4. The Pn, Gs and Tr values of the CK, W, Si and WSi treatments all decreased gradually with increasing Cd concentration. Compared with the CK, the Pn, Gs and Tr values were increased by 6.5–9.4%, 10.0–16.6% and 2.1–16.8% under the Si treatment; The values of Pn, Gs and Tr decreased by 20.5–27.9%, 8.6–26.8% and 13.3–23.3% under the W treatment; The values of Pn, Gs and Tr decreased by 13.1–21.2%, 3.7–22.3% and 2.2–13.7% under the WSi treatment. The values of Pn, Gs and Tr showed a trend of Si > CK > WSi > W. The Ci values showed significant differences among treatments and at different Cd concentrations. The Ci values gradually increased under the CK and Si treatments and decreased under the WSi treatment as the Cd concentration increased.

### 2.5. Synergistic Effects of Water Management and Silicon Foliar Spraying on MDA, SOD and POD Content in Rice Leaves

**Figure 5:**
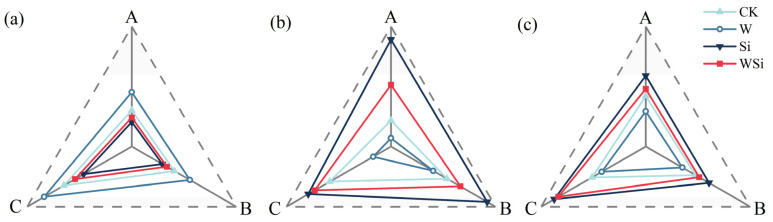
Radar plots of the synergistic effects of water management and silicon foliar spraying on MDA, SOD and POD contents in rice leaves ((**a**) malondialdehyde (MDA); (**b**) superoxide dismutase (SOD); (**c**) peroxidase (POD)). A, B and C represent the actual measured Cd content in soil after the addition of exogenous Cd at 0.20 mg·kg^−1^, 0.60 mg·kg^−1^ and 1.60 mg·kg^−1^, respectively.

The synergistic effects of water management and silicon foliar spraying on malondialdehyde (MDA), superoxide dismutase (SOD) and peroxidase (POD) content in rice leaves are shown in Figure 5. There was a gradual increase in MDA and a gradual decrease in SOD and POD. At the three Cd concentrations, compared to the CK treatment, the W treatment increased MDA content by 11.9–13.4% and decreased SOD and POD contents by 6.7–20.6% and 6.5–9.5%, respectively. The Si and WSi treatments reduced the MDA content and increased the SOD and POD contents. Following the Si treatment, the MDA content decreased by 8.7–12.7%, and the SOD and POD contents increased by 10.2–41.1% and 6.5–18.1%, respectively. Under the WSi treatment, the MDA content decreased by 5.3–7.2%, and the SOD and POD contents increased by 6.5–18.1% and 2.6–22.4%, respectively. The MDA content showed a trend of W > CK > WSi > Si, while the SOD and POD contents showed the opposite trend, that is, Si >WSi > CK > W.

## 3. Discussion

### 3.1. Synergistic Effects of Water Management and Silicon Foliar Spraying on Cd Content in Rice Organs and on Rice Yield

As the Cd concentration increased, the Cd content in every rice organ gradually increased, as did rice yield. The accumulation of Cd in rice affects its ultrastructure and inhibits its normal growth, thereby reducing its yield. At concentrations A and B, the continuous flooding treatment used in this study increased the Cd content in the roots, stems and leaves and decreased the Cd content in the husks and brown rice. This is inconsistent with the results of previous studies on the reduction in Cd content in rice roots via continuous flooding treatment [22]. The pH value of soil under continuous flooding treatment increases, promoting the adsorption of Cd by soil colloids and reducing the content of exchangeable Cd in the soil [23]. Some studies have shown that the soil pH affects the bioavailability of Cd in soil and the transport coefficient of Cd in various parts of rice plants. When the soil pH is high, more Cd accumulates in the root [24]. At concentration C, the continuous flooding treatment resulted in a significant reduction in Cd content in the roots, stems, leaves, cob, husks and brown rice. Because the continuous flooding treatment inhibited Cd dissolution in the soil, it promoted the formation of iron films and substantially reduced the transfer of Cd from the iron films to the roots of rice, resulting in reduced Cd uptake and accumulation by rice [25]. Continuous flooding treatment also has the potential to significantly reduce the biological efficacy of Cd in soil [26]. Moisture status has been shown to have a significant effect on rice yield, with continuous flooding treatments reducing rice yield throughout the reproductive period of rice [23]. This is because conventional intermittent flooding treatment can effectively improve soil redox and permeability, which not only facilitates the growth of soil micro-organisms and the roots of rice but also promotes organic matter decomposition and nutrient uptake, leading to an increase in rice biomass [27]. Conventional intermittent flooding is also beneficial for stimulating rice growth and has a compensatory rehydration effect following rice drought, when some degree of water deficit may increase rice yield [28]. Although continuous flooding treatment can significantly inhibit Cd uptake, it can also decrease rice yield.

At concentration A, Si treatment significantly increased the Cd content in the roots, leaves and cob and significantly reduced the Cd content in the husks and brown rice. At concentration B, the Si treatment significantly increased the Cd content in the stems and cob and significantly decreased the Cd content in the husks. At concentration C, the Si treatment significantly increased the Cd content in the leaves and cob and significantly reduced the Cd content in the husks and brown rice. For all three Cd concentrations, the Si treatment had no significant effect on rice yield. Some studies have shown that Si foliar spraying not only increases rice yield [29] but also reduces the accumulation of Cd in brown rice [30]. The node in the rice stem is the central organ for Cd transfer from the xylem to the phloem [31]. This is especially true for the first node, which extends from top to bottom in rice, showing the strongest cadmium enrichment capacity [23]. In this experiment, it is possible that spraying Si on the leaves increased the cadmium enrichment capacity of the nodes, thereby increasing the Cd content in rice stems. Si is beneficial for rice. The silicon sprayed on the leaves can be rapidly absorbed by the rice in the form of mono-silicon acid [18] and can form silicified cells in the rice epidermis, stem, leaf sheath and vascular tissue [32]. It can also form a negative chelate with the hemicellulose on the cell wall [33], thereby increasing the adsorption and Cd barrier [34]. In addition, Si-mediated mechanisms for reducing the toxicity of heavy metals in plants include (1) complexation and coprecipitation of heavy metals with Si, (2) stimulation of the antioxidant system and (3) enhanced photosynthesis efficiency [35]. Foliar spraying of Si alleviates the toxic effect of Cd on rice by increasing the Cd adsorption and the Cd barrier of Cd, improving photosynthesis, thereby increasing the yield. Foliar spraying of Si can not only reduce the absorption of Cd by brown rice but also promote photosynthesis and increase yield.

At all three Cd concentrations, the WSi treatment resulted in the lowest Cd content in brown rice compared with the W and Si treatments and did not affect rice yield. The WSi treatment had the best reduction effect on Cd. This is consistent with previous research results, indicating that water management and foliar spraying can achieve a good effect in terms of reducing Cd [36,37]. In conclusion, the W, Si and WSi treatments all significantly decreased the Cd content of brown rice, but the W treatment significantly reduced rice yield. The Si treatment increased rice yield, but its Cd reduction effect was not significant. Compared with water management and Si foliar spraying alone, WSi treatment was an effective means of ensuring rice yield while reducing Cd.

### 3.2. Synergistic Effects of Water Management and Silicon Foliar Spraying on the Cd Translocation Factor of Rice Organs

This study showed that continuous flooding treatment had a significant effect on Cd translocation in rice. At concentrations A and C, the W treatment reduced brown rice Cd content by increasing stem-to-leaf transport and by decreasing root-to-stem and leaf-to-brown rice transport. It enriched Cd in the roots, stems and leaves and reduced Cd translocation to brown rice, thereby reducing the Cd content in brown rice. This is consistent with previous research results [38]. Flooding treatment is an effective measure to reduce the levels of exchangeable Cd found in soil [39]. Under flooded conditions, some reducing bacteria, such as iron-reducing bacteria and sulfur-reducing bacteria, can promote the conversion of sulfate in soil to sulfide or divalent sulfur ions and can combine with Cd^2+^ to form insoluble Cd, which may reduce the mobility and bioavailability of Cd in soil [14]. Continuous flooding treatment can significantly reduce the content of Cd in the iron film of rice and in rice organs [13]. Therefore, water management can regulate the transport of Cd in rice and help to reduce the Cd content in grains. Si foliar spraying significantly reduced TF_root−stem_, TF_stem−brown rice_ and TF_leaf−brown rice_ and significantly increased TF_stem−leaf_. This is consistent with previous research results. Si foliar spraying inhibits the transport of Cd from root to stem and from the stem to brown rice and increases the transport of Cd from stem to leaf [40]. Researchers found that in acidic soil, Si foliar spraying can reduce the transfer coefficient of Cd from the stem to brown rice, thereby reducing the Cd content in brown rice [21]. Si foliar spraying can be assimilated into monomeric silicon acid by rice, which is mainly stored in rice leaves through the formation of a double cuticle layer, reducing the transport of Cd in rice [41]. In addition, Si foliar spraying results in the formation of a Si–Cd complex with Cd^2+^ in rice leaves, promotes the coprecipitation of Cd on the cell wall, reduces the proportion of Cd in the plastid and inhibits the transport of Cd [21]. The beneficial effects of Si are mainly associated with its high deposition in plant tissues, which may enhance their strength and rigidity, as well as improve their ability to overcome adverse types of stress [42].

The WSi treatment significantly increased TF_stem−leaf_ and significantly decreased TF_root−stem_ and TF_leaf−brown rice_. The continuous flooding and Si foliar spraying treatments had significant synergistic effects on the translocation of Cd to rice brown rice and were responsible for a significant decrease in Cd content in brown rice.

### 3.3. Synergistic Effects of Water Management and Silicon Foliar Spraying on SPAD Values of Rice Leaves

Chlorophyll is the primary pigment for photosynthesis in plants, and changes in its content are a direct reflection of the strength of photosynthesis [43]. The SPAD of rice leaves decreased with increasing Cd concentration. Cd inhibits chlorophyll biosynthesis [44] and interferes with the uptake of Ca, Mg, K, P and other elements [45], resulting in lower chlorophyll content [46]. The W treatment significantly reduced the SPAD value of compared with the CK treatment and had a more negative effect on chlorophyll synthesis. Si foliar spraying increased the SPAD value of rice, indicating that Si could alleviate the inhibitory effect of Cd stress on chlorophyll synthesis, thereby facilitating photosynthesis in rice. This is because Si foliar spraying promotes the activation of photosystem I (PSI) and photosystem II (PSII) by reducing the uptake of Cd and restoring photosynthesis [30]. In addition, Si not only facilitates the formation of silicified cells on the leaf surface but also enhances photosynthesis in leaves by improving the formation of endocysts in the chloroplasts of vascular sheath cells [47]; this may be why Si application can increase the SPAD value of rice leaves. The effect of the WSi treatment on the SPAD values was not significant, and the continuous flooding treatment in concert with Si foliar spraying did not significantly reduce the SPAD values of rice leaves.

### 3.4. Synergistic Effects of Water Management and Silicon Foliar Spraying on Photosynthesis in Rice

Photosynthesis is highly sensitive to Cd stress, and Pn is a determinant of rice yield [48]. Cd stress significantly reduced Gs and Tr in rice leaves, leading to a significant reduction in Pn [49], which resulted in yield reduction. The causes of Pn reduction can be divided into stomatal and non-stomatal factors [50], and Ci is an important indicator that can be used to distinguish between the two. The stomatal factor refers to a decrease in Pn with decreasing Ci, and the non-stomatal factor refers to an increase in Pn with decreasing Ci [51]. Pn decreased with a decrease in Ci under the CK and Si treatments, indicating that the primary reason for the decline in photosynthetic rate was a stomatal factor. On the other hand, the continuous flood treatment resulted in a decrease in Pn for the opposite reason, and both the W and WSi treatments reduced Pn with decreasing Ci, indicating that a non-stomatal factor was the primary cause of the decline in the photosynthetic rate. This study showed that the W treatment reduced Pn, Gs and Tr. In agreement with the results of the present study, a continuous flooding treatment caused damage to the photosynthetic machinery by reducing the content of Pn and chlorophyll in [15]. Pn, Gs and Tr were increased following Si foliar spraying. Ci and Tr are limiting factors for CO_2_ diffusion and carbon fixation; ribulose 1, 5–bisphosphate carboxylase oxygenase (RuBisCO), the rate-limiting enzyme for carbon assimilation, plays a decisive role in Pn and can mitigate the toxic effects of Cd stress on plants by increasing the carboxylation efficiency of RuBisCO [52]. Si foliar spraying of the leaves increases Gs and Tr, which, in turn, increases Pn, accelerating the effective carbon assimilation period of rice leaves, ultimately accelerating the accumulation of photosynthetic products and increasing rice yield [49]. The WSi treatment also decreased the photosynthetic parameters but increased them relative to the W treatment, indicating that foliar spraying with Si plays a role in promoting photosynthetic recovery.

### 3.5. Synergistic Effects of Water Management and Silicon Foliar Spraying on MDA, SOD and POD Content in Rice Leaves

Cd stress increases reactive oxygen species (ROS), and the excessive accumulation of ROS results in cellular redox imbalance and disrupted signaling, leading to growth inhibition and cell damage [53]. SOD and POD are metabolic enzymes that play a critical role in maintaining the balance of ROS. The SOD and POD activities of rice leaves under the W treatment were lower than those under the CK treatment, indicating that the flooding treatment induced a greater increase in ROS and intensified growth inhibition and cell damage, which was consistent with the result of reduced rice yield under the W treatment. Si foliar spraying enhanced the SOD activity of rice under different Cd stress concentrations, suggesting that Si may scavenge excess ROS that are produced due to Cd stress. This is consistent with the results of many studies, such as the sustained increase in SOD activity in rice roots [54] and leaves [55] under Zn stress following Si application and in wheat leaves under Cd stress [56]. Under Cd stress, POD activity was reduced in leaves but increased under Si foliar spraying. This may be due to the enhanced ability of non-enzymatic antioxidants, such as glutathione (GSH), non–protein thiol (NPT) and ascorbic acid (AsA) to remove ROS in a non-enzymatic manner [57,58,59]. Alternatively, this result could be due to the enhanced activity of other oxidant enzymes such as ascorbate peroxidase (APX) and SOD [60]. This is consistent with the results of other studies, such as the addition of Si increasing POD in cotton leaves [61] under Zn stress, in peanut [62] under Al stress and in rice [63] under Cd stress. The WSi treatment increased the SOD and POD contents of rice leaves under Cd stress, indicating that Si foliar spraying could improve the negative effects of flooding treatment on rice and promote normal growth.

MDA is a major product of lipid peroxidation, and changes in its content occur in response to the degree of lipid peroxidation and the relative permeability of cellular membranes in response to the degree of membrane damage, both of which have been shown to negatively correlate with plant stress resistance [64]. The rice leaf MDA content increased with increasing Cd stress concentration. The MDA content of rice leaves under the W treatment was greater than that under the CK treatment, indicating that the effect of the continuous flooding treatment on lipid peroxidation in leaves was more severe than that of Cd stress and that the flooding treatment was detrimental to antioxidant enzyme (SOD and POD) activity in rice. This indicates that the antioxidant capacity of rice could not effectively scavenge ROS generated under continuous flooding conditions, and lipid peroxidation took place in the leaves, resulting in the excessive accumulation of MDA [65]. The MDA content in the rice leaves decreased following Si foliar spraying, indicating that Si mitigated Cd-induced oxidative stress and played a protective role in Cd-induced lipid peroxidation. Rice was able to mitigate the toxicity of Cd through the foliar absorption of Si, thereby reducing the MDA content of rice and increasing its antioxidant capacity. This is consistent with the results of a study wherein rice and peanut were subjected to heavy metal stress [66,67]. In addition, Si reduced the permeability of the plasma membrane and membrane lipid peroxidation, maintaining the integrity and functional stability of the cell membrane [18]. MDA content was reduced by the WSi treatment, indicating that Si foliar spraying mitigated cell membrane damage caused by the continuous flood treatment.

## 4. Materials and Methods

### 4.1. Test Station and Materials

A rice pot experiment was conducted at the Guiyang Comprehensive Test Station of the Guizhou Academy of Agricultural Sciences, Guizhou, China (106°39′20″ E, 26°29′59″ N). The rice cultivar used in the experiment was “Jingliangyou 534”. The exogenous Cd added was CdCl_2_ (Aladdin, Shanghai, China). In the Si foliar spraying of “Jianggeling” rice foliar silicon fertilizer (Foshan Ironman Environmental Technology Co., Ltd., Foshan, China), the active ingredient was primarily high-purity SiO_2_ sols (Si ≥ 85 g·L^−1^, pH = 5.0–7.0) at a concentration of 2.5 g·L^−1^. One spray was administered at the jointing stage and one at the heading stage, at 18:00–19:00 p.m. The basic physical and chemical indicators of soils are shown in Table 2.

### 4.2. Experimental Design and Treatment

**Figure 6:**
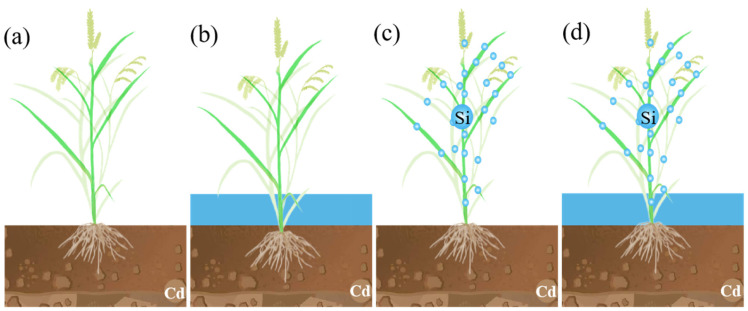
Diagram of the potting experiment ((**a**) conventional intermittent flooding + no Si foliar spraying (CK); (**b**) continuous flooding throughout the growth stage + no Si foliar spraying (W); (**c**) conventional intermittent flooding + Si foliar spraying; (**d**) continuous flooding throughout the growth stage + Si foliar spraying (WSi)).

The experiment was set up with three additions of exogenous Cd concentrations, namely A (0), B (0.20 mg·kg^−1^) and C (0.40 mg·kg^−1^); these were mixed with air-dried soil prior to rice planting, filled with water until they reached saturation and allowed to equilibrate for 30 s. The soil background value was 0.20 mg·kg^−1^, and the actual measured values of soil Cd content at maturity after Cd addition were 0.20 mg·kg^−1^ (A), 0.60 mg·kg^−1^ (B) and 1.60 mg·kg^−1^ (C). The four treatments were: conventional intermittent flooding + no Si foliar spraying (CK), continuous flooding throughout the growth stage + no Si foliar spraying (W), conventional intermittent flooding + Si foliar spraying (Si) and continuous flooding throughout the growth stage + Si foliar spraying (WSi) (Figure 6). There were 12 treatments in this experiment, with each treatment repeated 3 times.

### 4.3. Measurement Methods

#### 4.3.1. Determination of Cd Content

The Cd content of rice was determined using the ICP–OES software package (Thermo Fisher Scientific, Waltham, MA, USA) [68]. We weighed 200 mg of the rice sample and added 5 mL of nitric acid (HNO_3_). The sample was digested using a 120 °C graphite digestion instrument for 2 h until there was no sediment in the digestion tank. Then, the temperature was adjusted to 150 °C to evaporate the acid. The sample was removed and allowed to cool, diluted to a volume of 50 mL in a volumetric flask, filtered and analyzed via ICP–OES.

#### 4.3.2. Determination of Relative Chlorophyll Content

The relative chlorophyll content of the leaves was determined using SPAD-502 Plus (Minolta, Tokyo, Japan) [69]. Three uniformly growing rice plants were selected, and the midpoint of the leaf was determined. Each position was determined 6 times, and the average value was taken as the SPAD value.

#### 4.3.3. Determination of Photosynthetic Parameters

The parameters of photosynthesis during the tasseling of rice were determined using a GFS-3000 (Heinz Walz GmbH, Bavaria, Germany) [70]. The CO_2_ concentration was set at 400 μmol·mol^−1^, the light intensity at 1200 μmol·m^−2^·s^−1^ and the air flow rate at 0.5 L·min^−1^ on a sunny day from 10:00 a.m. to 12:00 p.m. The net photosynthetic rate (Pn), transpiration rate (Tr), stomatal conductance (Gs) and intercellular CO_2_ concentration (Ci) of rice were determined on uniformly grown rice leaves at 25 °C and 70% relative humidity.

#### 4.3.4. Determination of MDA, SOD and POD Contents

We weighed 100 mg fresh rice leaves, ground them to a homogenate in a mortar using liquid nitrogen and placed the homogenate in a 4 mL centrifuge tube, followed by the addition of 1 mL of 0.05 mol·L^−1^ phosphate buffer (pH 7.8); then, we adjusted to volume to 4 mL. We mixed the solution well using a vortexer, placed in a frozen high-speed centrifuge at 4 °C and 10,000 r·min^−1^ for 10 min and transferred the supernatant into a refrigerator at 4 °C. To determine the contents of SOD, POD and MDA in rice leaves, a photochemical reduction method using nitrogen blue tetrazolium (NBT) [71], the guaiacol method and the thiobarbituric acid (TBA) method were employed, respectively, all of which used enzymatic activity kits from Wuhan Purity Biological Co. (Wuhan, China).

### 4.4. Statistical Analysis

The translocation factor (TF) of Cd in rice was calculated as follows [72].

$${\mathrm{TF}}_{\mathrm{root}-\mathrm{stem}}=\;\mathrm{Cd}\;\mathrm{content}\;\mathrm{in}\;\mathrm{stem}/\mathrm{Cd}\;\mathrm{content}\;\mathrm{in}\;\mathrm{root};$$

$${\mathrm{TF}}_{\mathrm{stem}-\mathrm{leaf}}=\;\mathrm{Cd}\;\mathrm{content}\;\mathrm{in}\;\mathrm{leaf}/\mathrm{Cd}\;\mathrm{content}\;\mathrm{in}\;\mathrm{stem};$$

$${\mathrm{TF}}_{\mathrm{root}-\mathrm{stem}}=\mathrm{Cd}\;\mathrm{content}\;\mathrm{in}\;\mathrm{brown}\;\mathrm{rice}/\mathrm{Cd}\;\mathrm{content}\;\mathrm{in}\;\mathrm{stem};$$

$${\mathrm{TF}}_{\mathrm{root}-\mathrm{stem}}=\mathrm{Cd}\;\mathrm{content}\;\mathrm{in}\;\mathrm{brown}\;\mathrm{rice}/\mathrm{Cd}\;\mathrm{content}\;\mathrm{in}\;\mathrm{leaf}.$$

The results obtained in this study were expressed as the mean value ± standard deviation of 3 repetitions. Statistical analysis was performed using SPSS 24.0 software (SPSS Inc., Chicago, IL, USA). One-way analysis of variance (ANOVA) with Fisher’s least significant difference (LSD) test was used to evaluate the significance of the effects of different levels on measured parameters.

## 5. Conclusions

In this study, we investigated the synergistic effects of water management and silicon foliar spraying on rice yield and Cd accumulation. Compared with the CK treatment, the W treatment reduced the chlorophyll content and photosynthesis in rice, and the Si treatment protected against the damage caused to photosynthetic structures. Si foliar spraying mitigated the adverse effects of continuous flooding treatment and Cd stress on rice by reducing the membrane permeability and MDA content and enhancing SOD and POD activities. The results show that, although the W treatment significantly reduced the Cd content of brown rice, it reduced the yield. Si treatment not only improved the rice yield but also reduced the Cd content in brown rice; however, the reduction was not significant. WSi treatment significantly reduced the Cd content in brown rice while ensuring a good rice yield. In conclusion, synergistic continuous flooding throughout the growth stage, combined with Si foliar spraying, is an effective means of reducing Cd accumulation in brown rice.

## Figures and Tables

**Table 1:** Synergistic effects of water management and silicon foliar spraying on Cd content in rice organs and on rice yield. There is no significant difference between values with the same letters in the table (*p* ≥ 0.05, Fisher’s LSD test). A, B and C represent the actual measured Cd content in soil after the addition of exogenous Cd at 0.20 mg·kg^−1^, 0.60 mg·kg^−1^ and 1.60 mg·kg^−1^, respectively.

Cd	Treatment	Cd Content in Various Parts of Rice (mg·kg^−1^)						Yield (kg·ha^−1^)
		Root	Stem	Leaf	Cob	Husk	Brown Rice	
A	CK	0.431 ± 0.039 d	0.063 ± 0.009 a	0.020 ± 0.002 c	0.027 ± 0.004 b	0.024 ± 0.003 ab	0.044 ± 0.010 a	4369.4 ± 157.6 ab
	W	0.591 ± 0.078 c	0.033 ± 0.005 b	0.030 ± 0.001 b	0.021 ± 0.003 b	0.028 ± 0.004 a	0.014 ± 0.002 b	3987.3 ± 151.6 c
	Si	0.918 ± 0.058 a	0.074 ± 0.004 a	0.026 ± 0.005 b	0.041 ± 0.005 a	0.017 ± 0.001 b	0.026 ± 0.011 b	4552.1 ± 116.9 a
	WSi	0.726 ± 0.084 b	0.035 ± 0.010 b	0.037 ± 0.002 a	0.023 ± 0.002 b	0.026 ± 0.006 a	0.014 ± 0.002 b	4115.5 ± 149.2 bc
B	CK	1.137 ± 0.153 b	0.071 ± 0.002 b	0.053 ± 0.003 ab	0.044 ± 0.005 b	0.048 ± 0.004 c	0.109 ± 0.019 a	4144.6 ± 76.6 a
	W	1.295 ± 0.202 b	0.078 ± 0.006 b	0.045 ± 0.002 b	0.049 ± 0.005 b	0.031 ± 0.009 a	0.099 ± 0.013 ab	3370.9 ± 152.7 b
	Si	1.259 ± 0.114 b	0.174 ± 0.022 a	0.057 ± 0.007 a	0.071 ± 0.004 a	0.028 ± 0.005 b	0.103 ± 0.011 ab	4242.5 ± 207.2 a
	WSi	1.844 ± 0.112 a	0.156 ± 0.018 a	0.049 ± 0.003 ab	0.070 ± 0.007 a	0.029 ± 0.007 b	0.079 ± 0.008 c	3615.6 ± 151.1 b
C	CK	2.639 ± 0.409 a	0.601 ± 0.040 a	0.102 ± 0.006 b	0.164 ± 0.013 b	0.118 ± 0.010 a	0.167 ± 0.011 a	3654.9 ± 234.2 ab
	W	1.874 ± 0.444 b	0.178 ± 0.016 c	0.082 ± 0.002 c	0.131 ± 0.005 c	0.054 ± 0.010 c	0.117 ± 0.004 c	3152.2 ± 156.6 c
	Si	2.931 ± 0.081 a	0.611 ± 0.026 a	0.129 ± 0.004 a	0.267 ± 0.018 a	0.078 ± 0.008 b	0.146 ± 0.008 b	3879.3 ± 229.5 a
	WSi	2.339 ± 0.224 ab	0.231 ± 0.005 b	0.100 ± 0.004 b	0.169 ± 0.008 b	0.052 ± 0.008 c	0.096 ± 0.015 d	3359.2 ± 182.1 bc

**Table 2:** Basic physical and chemical indicators of soils.

Property	Value
Soil type	Yellow soil
pH	6.58
Organic matter content (g∙kg^−1^)	115.01
Alkali hydrolysable N (mg∙kg^−1^)	49.25
Rapidly available P (mg∙kg^−1^)	5.96
Rapidly available K (mg∙kg^−1^)	153.90
Total Cd (mg∙kg^−1^)	0.20

## Data Availability

The data presented in this study are available upon request from the corresponding author.
